# Anti-Metabolic Dysfunction-Associated Hepatic Steatosis Effects of Pickering Emulsion-Encapsulated Curcumin via Gut Microbiota and Short-Chain Fatty Acids Modulation in High-Fat-Diet Mice

**DOI:** 10.3390/foods14234009

**Published:** 2025-11-22

**Authors:** Lisha Niu, Fengyang Wu, Yingxue Jiao, Chi Ren, Ying Shu, Yi Liu, Kaixuan Zhao, Weili Rao, Liwen Wang, Zhisheng Zhang, Wenhui Qi

**Affiliations:** 1 College of Food Science and Technology, Hebei Agricultural University, Lekai South Avenue, Baoding 071000, China; 15190272226@163.com (L.N.);; 2 Baoding Product Quality Supervision and Inspection Institute, Baoding 071000, China

**Keywords:** pickering emulsion-encapsulated curcumin, gut microbiota, short-chain fatty acids, metabolic dysfunction-associated hepatic steatosis

## Abstract

With lipid metabolism disorders becoming a global health issue, designing safe and effective prevention methods is crucial. Curcumin, a natural polyphenol compound, exhibits the ability to ameliorate lipid metabolism disorders. However, the bioavailability of curcumin is low, primarily due to its poor solubility, susceptibility to degradation, low absorption, and rapid metabolism. The bioavailability of curcumin is markedly enhanced when it is encapsulated within Pickering emulsions. This study investigated the ameliorative effect of curcumin encapsulated in Pickering emulsions on high-fat-diet-induced lipid metabolism disorders. We demonstrated that curcumin encapsulated in Pickering emulsions substantially prevented high-fat-diet-induced body gain, alleviated glucose intolerance, mitigated insulin resistance, and improved hepatic steatosis. Curcumin encapsulated in Pickering emulsions induced gut microbiota remodeling, characterized by an increased relative abundance of Bacteroidetes and a decreased relative abundance of Firmicutes. In particular, the relative abundance of *Akkermansia* was significantly increased. The changes in the gut microbiota of mice fed a high-fat diet, curcumin, or curcumin encapsulated in Pickering emulsions mice were correlated with lipid-related parameters in serum (triglycerides, total cholesterol, high-density lipoprotein cholesterol) and the generation of short-chain fatty acids. These findings indicated the basis of curcumin’s effects by modulating gut microbiota-short-chain fatty acids, offering valuable perspectives for developing it as a potential functional food component aimed at preventing and mitigating metabolic dysfunction-associated hepatic steatosis.

## 1. Introduction

Lipid metabolism plays an important role in modulating metabolic balance and human health. Excessive food intake, particularly high-calorie diets (e.g., high-fat and high-sugar diets), induces lipid metabolism disorders (LMDs) [1]. LMDs contribute to various diseases, including hyperlipidemia [2], obesity [3], non-alcoholic fatty liver disease [4], and diabetes [5]. Therefore, identifying food-derived bioactive substances to effectively prevent LMDs is greatly significant for promoting human health.

Curcumin (Cur) is a natural polyphenol that can ameliorate LMDs without side effects [6]. The recommended edible dosage of Cur is up to 12 g/day [7]. Curcumin has attracted widespread attention for its ability to alleviate cadmium-induced gut microbiota dysbiosis and lipid metabolic disorders by modulating the gut microbiota and their metabolites [8]. Curcumin modulates the gut microbial structure by reducing *Lactobacillus* and increasing *Unspecified_S24_7* and *Akkermansia*, which ameliorates LMDs [8]. Cur can also markedly ameliorate high-fat-diet (HFD)-induced liver steatosis and insulin resistance (IR) in obese mice [9]. One of the mechanisms by which it does this is altering gut microbial composition [9]. It also promotes the proliferation of short-chain fatty acids (SCFAs) producing bacteria and reduces endotoxin-producing *Desulfovibrio* [9]. Cur ameliorates HFD-induced LMDs by upregulating fibroblast growth factor 15 (FGF15) via the gut microbiota [10]. Therefore, investigating the correlation between Cur-regulated amelioration of LMDs and gut microbiota composition is of great importance. It is also crucial to explore the relationship with gut microbial metabolites.

It is well-known that Cur exerts beneficial effects on LMDs. However, the bioavailability of Cur is low, which is primarily attributable to its poor water solubility and low chemical stability [11]. Compared with conventional emulsions, Pickering emulsions (PEs) have been widely applied in the food industry due to their superior stability, biocompatibility, safety, cost-effectiveness, and wider applicability [12]. PEs have evolved into a promising new platform for the encapsulation and controlled release of Cur [13]. In vitro digestion and encapsulation in the Pickering emulsion stabilized by heat-treated gluten fibrin increases the bioavailability of Cur in the oil phase from 16.81% to 35.06% [14]. Similarly, the bioavailability of Cur in multi-stimuli-responsive nanoparticle-based PE (35.26%) is substantially higher than that in rapeseed oil (8.19%) [15]. The Cur bioavailability in the oil phase, initially 19.53%, is increased to 41.26% after encapsulation in PE stabilized with Chinese water chestnut-9% octenyl succinic anhydride [16]. These findings indicate that the bioavailability of Cur is significantly improved to varying degrees in vitro when encapsulated in PEs [12,13,14]. Most current studies focus on simulated gastrointestinal digestion systems, and in vivo evaluations regarding lipid metabolism disorders remain limited [11].

Therefore, this study aims to investigate the ameliorative effect of curcumin encapsulated in Pickering emulsions on high-fat-diet-induced LMDs. A high-fat-diet-induced lipid metabolism disorder mouse model was developed and curcumin encapsulated in Pickering emulsions was administered as the intervention. The obesity-related phenotypes and serum lipid metabolism-related parameters, hepatic steatosis, hepatic and adipose tissue histopathology, gut microbiota and metabolites, and short-chain fatty acids were systematically assessed. Through these analyses, we aimed to assess whether curcumin encapsulated in Pickering emulsions enhances efficacy relative to free curcumin and to clarify the mechanisms.

## 2. Materials and Methods

### 2.1. Materials, Reagents and Diets

Curcumin (Cur, 95.34%) was sourced from Chenguang Biotechnology Group Co., Ltd. (Batch Number: C1-0320-240524; Handan, China). Sulfonated nanocellulose whiskers (CNC-C) aqueous solution was purchased from Qihong Technology Co., Ltd. (Cat. No.: HN202519-01; Guilin, China). Beeswax was purchased from Changge Ruifengfang Beekeeping Industry Co., Ltd. (Xuchang, China); no catalog number was provided for this product. Fortune first-class soybean oil was purchased from COFCO Oils & Oilseeds Industry Co., Ltd. (Jingzhou, China); no catalog number was provided for this product.

Animal diets: Normal diet (ND, 10% energy from fat, Cat. No.: D12450B) and HFD (60% energy from fat, Cat. No.: D12492) were purchased from SPF Biotechnology Co., Ltd. (Beijing, China). The oil-water mixture containing Cur and the PE-Cur used in the experiment were prepared independently in the laboratory. The curcumin encapsulated in Pickering emulsions (PE-Cur) formulation used was selected based on previously established and validated characterization in our laboratory, which demonstrated uniform droplet size, good stability, and effective encapsulation efficiency. Therefore, this study focused on evaluating the in vivo effects of PE-Cur.

### 2.2. Animals and Experimental Design

**Figure 1:**
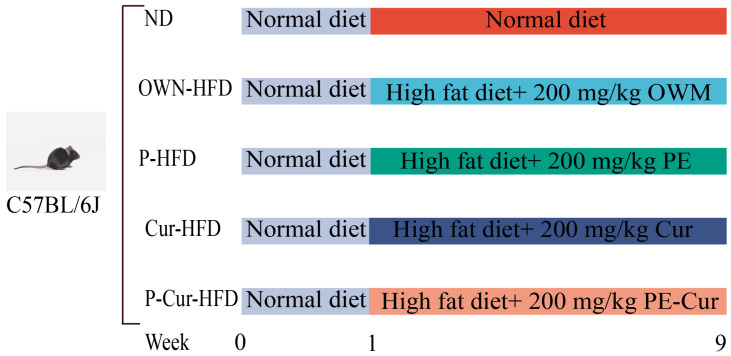
Animals and experimental design.

All animal procedures complied with the Guidelines for Ethical Review of Laboratory Animal Welfare in China and were approved by the Laboratory Animal Ethics Committee of Hebei Agricultural University (Approval No. 2025049). Four-week-old male C57BL/6J mice were housed in a standard environment with free access to food and water (temperature: 23 ± 2 °C, 12 h light/dark cycle). After one week of acclimatory feeding with the D12450B diet, the mice were randomly assigned to 5 groups (*n* = 8): normal diet group (ND); HFD supplemented with oil-water mixture group (OWM-HFD); HFD supplemented with the oil-in-water gel PE group (P-HFD); HFD supplemented with oil-water mixture containing Cur group (Cur-HFD, 200 mg/kg); and HFD supplemented with the oil-in-water gel PE encapsulating Cur group (P-Cur-HFD, 200 mg/kg) (Figure 1). The mice weight was recorded once a week. The experiment ended after 9 weeks. The mice were euthanized by cervical dislocation. Plasma was collected via eyeball enucleation. Plasma was centrifugated using an SLX-1024F centrifuge (Servicebio Technology Co., Ltd., Wuhan, China) (3000 r/min, 15 min, 4 °C), after which serum was collected. The weights of brown adipose tissue (BAT), liver, inguinal white adipose tissue (iWAT), and epididymal white adipose tissue (eWAT) were respectively recorded, and part of these tissues were placed in 4% paraformaldehyde solution (PFA) for histological analysis.

### 2.3. Oral Glucose Tolerance Test (OGTT) and Insulin Tolerance Test (ITT)

OGTT and ITT were performed as previously described [9]. Specifically, OGTT was conducted after mice was fasted for 12 h, while ITT was measured after a 2 h fast. Blood glucose levels were detected using an SE102 glucose analyzer (Sanno Bio-Sensing Co., Inc., Changsha, China). Subsequently, the area under the curve (AUC) was calculated.

### 2.4. Biochemical Assays

The concentrations of triglycerides (TG; Cat. No.: G2025), total cholesterol (TC; Cat. No.: G2027), low-density lipoprotein cholesterol (LDL-C; Cat. No.: G2032), high-density lipoprotein cholesterol (HDL-C; Cat. No.: G2031), alanine aminotransferase (ALT; Cat. No.: G1006), and aspartate aminotransferase (AST; Cat. No.: G1007) were determined using commercial kits (Servicebio Technology Co., Ltd., Wuhan, China) with a Chemray 420 automatic biochemical analyzer (Raysha Life Science & Technology Co., Ltd., Shenzhen, China). The levels of adiponectin (ADPN; Cat. No.: ml037840) and leptin (LEP; Cat. No.: ml063159) were assayed using ELISA kits (Shanghai Enzyme-linked Biotechnology Co., Ltd., Shanghai, China) with an Epoch enzyme label detector (BioTeK Instruments, Inc., Winooski, VT, USA).

### 2.5. Oil Red O Staining

Livers fixed in 4% PFA were paraffin-embedded using a JB-P5 embedding apparatus (Junjie Electronics Co., Ltd., Wuhan, China). Paraffin-embedded liver tissues were sectioned with a CRYOSTARNX5 slicer (Thermo Fisher Scientific Co., Ltd., Shanghai, China), and the sections were stained with Oil Red O. The stained samples were examined using an ECLIPSE Ci light microscope (Nikon, Tokyo Metropolis, Japan), and the area of adipocytes in the liver was quantified using ImageJ 1.52V (National Institutes of Health, Bethesda, MD, USA).

### 2.6. Hematoxylin and Eosin Staining

Liver, BAT, eWAT and iWAT fixed in 4% PFA were processed for paraffin embedding, cut into sections, and subjected to hematoxylin–eosin (H&E) staining. The stained samples were then examined and analyzed under the light optical microscope.

### 2.7. 16S rDNA Sequencing

Bacterial genomic DNA was extracted from cecal contents following the instructions of the E.Z.N.A.^®^ soil DNA kit (Cat. No.: D5625; Omega Biotek, Norcross, GA, USA). The V3-V4 variable region of 16S rRNA was amplified with the primer 338F (5′-ACTCCTAC-GGGAGGCAGCAG-3′), and the downstream primer 806R (5′-GGACTACHVGGGTWTCTAAT-3′) with Barcode sequences on a T100 Thermal Cycler PCR machine (BIO-RAD, Hercules, CA, USA). Sequencing was carried out on the Illumina Nextseq 2000 system (Meiji Biomedical Technology Co., Ltd., Shanghai, China), and the sequence data was analyzed via the Meggie Bio Cloud platform (https://cloud.majorbio.com) (accessed on 22 March 2025). Taxonomic assignments were performed using the SILVA database. The term “un-classified_f_” denotes taxa that could be assigned at the family level but were not sufficiently resolved to the genus level based on SILVA reference sequences.

### 2.8. Assessment of SCFAs Concentrations

The quantification of SCFAs in the cecal content was performed as previously described [17]. Twenty milligrams of cecal contents were mixed with 800 microliters of 0.5% phosphoric acid aqueous solution (containing 10 micrograms per milliliter of 2-ethylbutyric acid as an internal standard) in a centrifuge tube. Samples were first ground in a Wonbio-96c multi-sample cryogenic grinder (Wanbai Biotechnology Co., Ltd., Shanghai, China) (3 min, 50 Hz), followed by ultrasonic treatment in an SBL-10DT ultrasonic thermostatic cleaning machine (Ningbo Xinzhi Biotechnology Co., Ltd., Ningbo, Zhejiang, China) (30 °C, 60 kHz, 3 W/cm^2^, 10 min). Subsequently, they were centrifuged using a Centrifuge 5430R cryogenic centrifuge (Eppendorf, Hamburg, Germany) (4 °C, 13,000× *g*, 15 min). The supernatant was extracted with n-butanol, vortexed for 10 s, sonicated (4 °C, 60 kHz, 3 W/cm^2^, 10 min), and centrifuged (4 °C, 13,000× *g*, 5 min), and the final supernatant was transferred into an injection vial.

Gas chromatography mass spectrometry was employed to analyze the samples. The temperature programming conditions were set as follows: started at 80 °C, ramped to 120 °C at 20 °C/min and then gradually to 160 °C at 5 °C/min, followed by a 220 °C hold for 3 min with a post-run. The mass spectrometry conditions were as follows: electron impact ion source at 230 °C, quadrupole temperature at 150 °C, transfer line temperature at 230 °C, electron energy at 70 electron volts, and scanning mode in selected ion monitoring. SCFAs quantification was performed using a six-point standard calibration curve.

### 2.9. Statistical Analysis

Data were expressed as mean ± relative standard deviation (RSD). The number of biological replicates is shown in the caption of the results graph. Statistical analyses were performed using SPSS software 26 (SPSS Inc., Chicago, IL, USA). Differences between different treatment groups were compared using one-way analysis of variance (ANOVA). *p* < 0.05 was considered statistically significant. The data were graphed using GraphPad Prism 10.1.2 software (San Diego, CA, USA). Before performing ANOVA, the Shapiro–Wilk test was used to assess the normality of the data distribution, and Levene’s test was applied to evaluate the homogeneity of variances. Gut microbial Alpha diversity was calculated through the Meggie Bio-Cloud Platform (https://cloud.majorbio.com) (accessed on 22 March 2025). Alpha diversity among groups was compared using the Wilcoxon rank-sum test. Community composition similarity was analyzed using Bray-Curtis-based principal coordinate analysis (PCOA), and non-metric multidimensional scaling (NMDS) was applied to illustrate differences in microbial community profiles among groups. Linear discriminant analysis effect size (LEFSE) (LDA score > 2 and *p* < 0.05) was conducted to determine differentially abundant bacterial taxa across phylum to genus among groups. Spearman’s correlation analysis was conducted to determine correlations between gut microbiota and serum lipid-related parameters, as well as between gut microbiota and SCFAs.

## 3. Results

### 3.1. PE-Cur Attenuated High-Fat-Diet-Induced Obesity Phenotype

**Figure 2:**
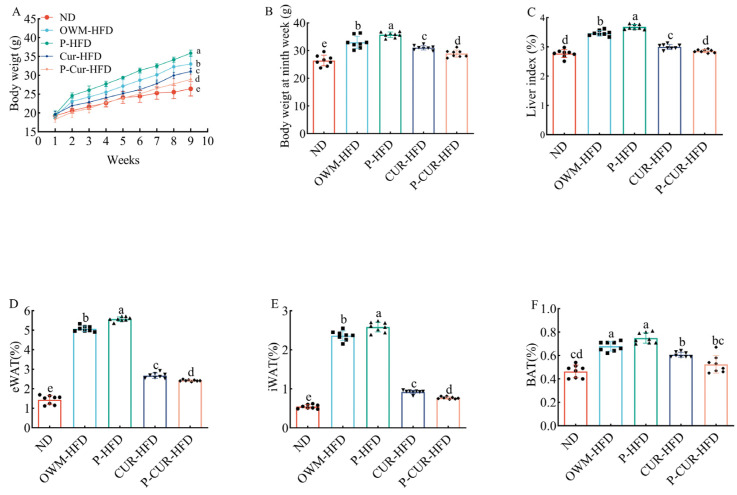
Effects of PE-Cur on the body weight, liver index, and fat indices in HFD mice. (**A**) Body weight curve; (**B**) body weight in the ninth week, highlighting the between-group differences to demonstrate the effect of each treatment relative to the ND; (**C**) liver index; (**D**) epididymal white adipose tissue (eWAT) index; (**E**) inguinal white adipose tissue (iWAT) index; (**F**) brown adipose tissue (BAT) index. Data are presented as mean ± RSD (n = 8). (a, b, c, d, e values with different superscripts differ significantly at *p* < 0.05).

Compared with the ND group, the HFD groups (OWM-HFD and P-HFD) showed higher body weight, liver index, and adiposity indices (including eWAT, iWAT, and BAT) after 9 weeks of HFD feeding. The Cur intervention groups (Cur-HFD and P-Cur-HFD) mitigated these increases (Figure 2A–F, *p* < 0.05). Further comparisons between the HFD subgroups revealed that the P-HFD group showed higher body mass, liver index, and adiposity indices than the OWM-HFD group (Figure 2A–E, *p* < 0.05). Among the Cur intervention subgroups, the P-Cur-HFD group had lower body weight, liver index, and adiposity indices relative to the Cur-HFD group (Figure 2A–E, *p* < 0.05). No significant differences in liver index and BAT index were detected between the P-Cur-HFD group and the ND group (Figure 2C,F, *p* > 0.05).

### 3.2. PE-Cur Alleviated Glucose Intolerance and IR

**Figure 3:**
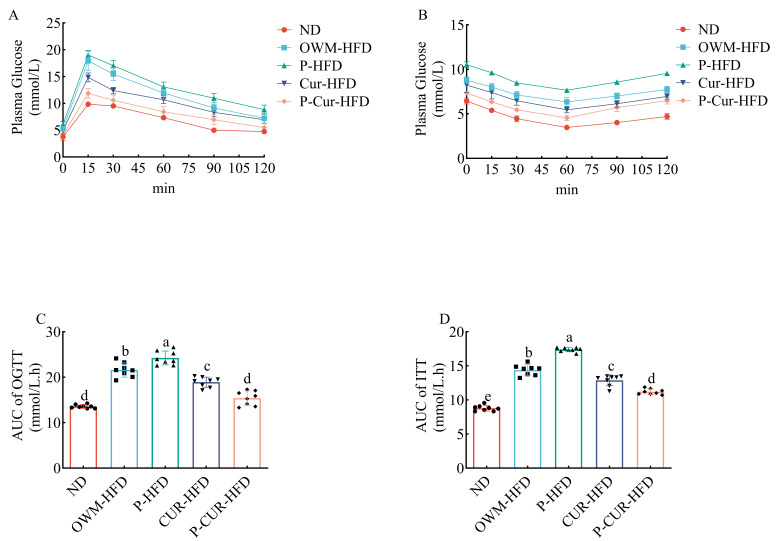
PE-Cur improved glucose intolerance and insulin resistance in HFD mice. (**A**) Blood glucose levels for OGTT at the 8th week; (**B**) blood glucose levels for ITT at the 9th week; (**C**) area under the OGTT curve at the 8th week; (**D**) area under the curve of ITT at the 9th week. Data are presented as mean ± RSD (*n* = 8). (a, b, c, d, e values with different superscripts differ significantly at *p* < 0.05).

After glucose intake, the blood glucose first increased, then decreased, and finally stabilized (Figure 3A). After the injection of fasting insulin, the blood glucose level in mice first decreased, then increased, and finally stabilized (Figure 3B). At each measurement time point, the serum glucose values were ordered as follows: ND < P-Cur-HFD < Cur-HFD < OWM-HFD < P-HFD. The AUC of blood glucose was considerably higher in the HFD groups (OWM-HFD, P-HFD) than in the ND and Cur intervention (Cur-HFD, P-Cur-HFD) groups (Figure 3C,D, *p* < 0.05). Compared with the OWM-HFD group, the AUC of blood glucose in the P-HFD group was markedly higher (Figure 3C,D, *p* < 0.05). While the AUC of blood glucose in the P-Cur-HFD group was significantly lower than that in the Cur-HFD group (Figure 3C,D, *p* < 0.05).

### 3.3. PE-Cur Alleviated Serum Metabolic Disorders in LMD Mice

**Figure 4:**
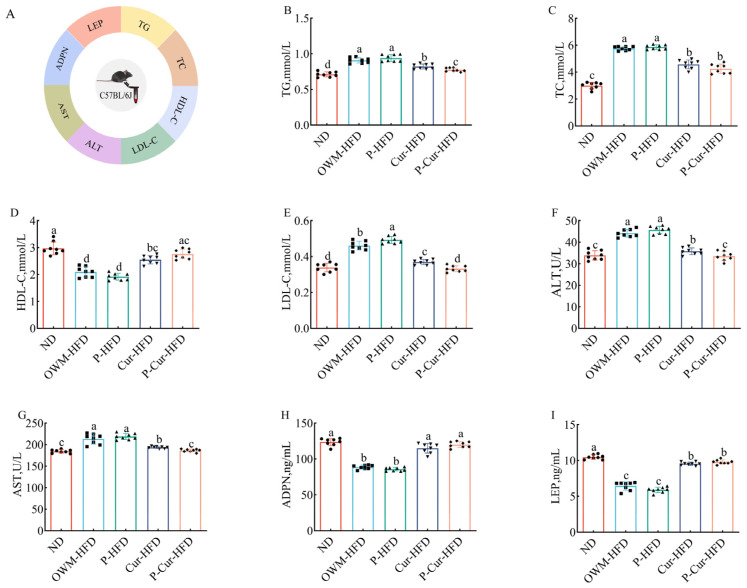
PE-Cur intervention alleviates abnormal serum factors affected by a high-fat diet. (**A**) Schematic diagram of the measured indicators in serum; (**B**) triglyceride (TG); (**C**) total cholesterol (TC); (**D**) high-density lipoprotein cholesterol (HDL-C); (**E**) low-density lipoprotein cholesterol (LDL-C); (**F**) alanine aminotransferase (ALT); (**G**) aspartate transaminase (AST); (**H**) adiponectin (ADPN); (**I**) leptin (LEP). Data are presented as mean ± RSD (*n* = 8). (a, b, c, d values with different superscripts differ significantly at *p* < 0.05).

To investigate the effect of the PE-Cur on serum lipid-related metabolic parameters in HFD mice, the serum levels of TC, TG, HDL-C, LDL-C, AST, ALT, ADPN, and LEP were measured (Figure 4A). As illustrated in Figure 4B–I, in contrast to the ND group and Cur intervention groups (Cur-HFD, P-Cur-HFD), the HFD groups (OWM-HFD, P-HFD) presented higher levels of TG, LDL-C, TC, ALT, and AST, along with lower levels of HDL-C, ADPN, and LEP (*p* < 0.05). Relative to the Cur-HFD group, the P-Cur-HFD group showed lower levels of TG, LDL-C, ALT, and AST (*p* < 0.05).

### 3.4. PE-Cur Alleviated Hepatic Lipid Accumulation

**Figure 5:**
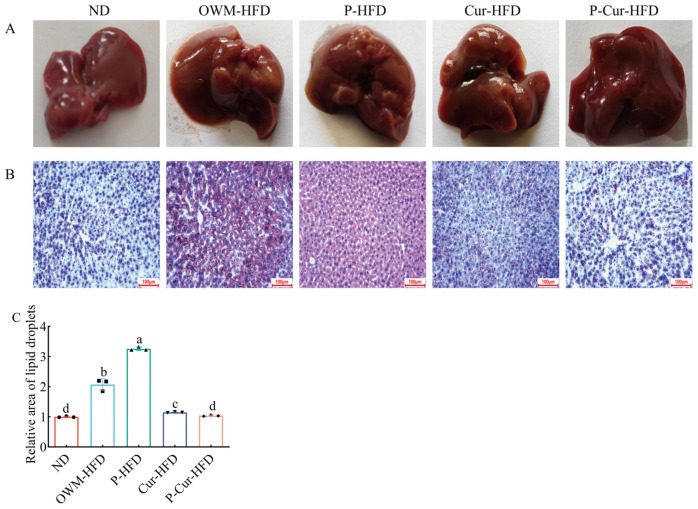
PE-Cur improves hepatic steatosis affected by HFD. (**A**) Livers of different treatment groups; (**B**) oil red O-stained sections of liver from different treatment groups (scale is 100 μm; red: lipids; blue: nuclei; white: background); (**C**) relative area of lipid droplets in the liver. Data are presented as mean ± RSD (*n* = 3). (a, b, c, d values with different superscripts differ significantly at *p* < 0.05).

As presented in Figure 5A, the white fat accumulation around the liver was higher in HFD groups (OWM-HFD, P-HFD) than that in the ND group and Cur intervention groups (Cur-HFD, P-Cur-HFD). Minor white fat accumulation was observed around the liver in the Cur intervention groups, a finding comparable to that in the ND group. The results in Figure 5B demonstrated that hepatic lipid droplets were higher in the HFD groups relative to that in the ND group and Cur intervention groups. Relative to the OWM-HFD group, lipid droplet accumulation in the liver was markedly higher in the P-HFD group. While the count of lipid droplets in the liver of the P-Cur-HFD group was lower than that in the Cur-HFD group, it was more comparable to that in the ND group. As shown in Figure 5C, the relative hepatic lipid droplet area displayed a trend consistent with the aforementioned results. The relative hepatic lipid droplets area in the HFD groups was markedly higher than that in the ND group and Cur intervention groups (*p* < 0.05). The relative hepatic lipid droplets area was lower in the P-Cur-HFD group compared with that in the Cur-HFD group (*p* < 0.05) and more comparable to that in the ND group (*p* > 0.05). The results indicated that HFD-induced hepatic fat accumulation led to hepatic steatosis. Cur alleviated hepatic fat accumulation. Moreover, the ameliorative effect of PE-Cur was more significant compared with that of Cur.

### 3.5. PE-Cur Improved Histopathological Changes in Liver, BAT, eWAT, and iWAT

**Figure 6:**
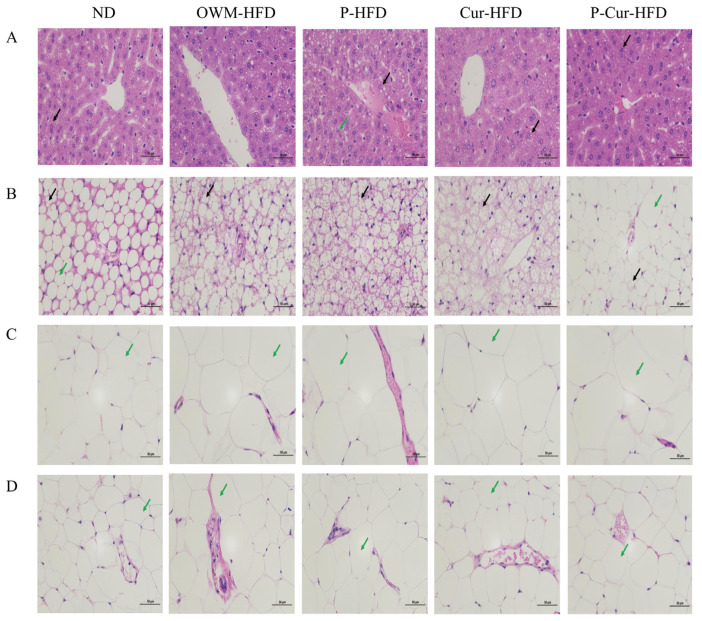
PE-Cur ameliorates histopathological changes in the liver, brown adipose tissue affected by HFD (BAT), epididymal white adipose tissue (eWAT), and inguinal white adipose tissue (iWAT). (**A**) Hematoxylin and eosin (H&E)-stained sections of liver; (**B**) hematoxylin and eosin (H&E)-stained sections of BAT; (**C**) hematoxylin and eosin (H&E)-stained sections of epididymal white adipose tissue (eWAT); (**D**) hematoxylin and eosin (H&E)-stained sections of inguinal white adipose tissue (iWAT). (**A**): black arrows: rounded vacuoles; green arrows: cytoplasmic laxity and light staining; (**B**–**D**): Black arrows: small lipid droplets dispersed in the cytoplasm; green arrows: large adipocyte volume; (scale is 50 μm).

HFD groups (OWM-HFD, P-HFD) showed more severe hepatic vacuolar degeneration (Figure 6A), greater lipid droplet accumulation in BAT (Figure 6B) compared with the ND group and Cur intervention groups (Cur-H, P-Cur-H). Additionally, disorganized adipose cell arrangement in BAT (Figure 6B), as well as cell hypertrophy in eWAT and iWAT (Figure 6C,D), were more obvious in the HFD groups. As shown in Figure 6A–D, the aforementioned pathological manifestations were significantly alleviated in the Cur intervention groups. Compared with the Cur-HFD group, the P-Cur-HFD group exhibited a more significant improvement. The results showed that HFD-induced fat accumulation in liver fat, BAT, eWAT, and iWAT caused histopathological alterations in hepatic and adipose tissues. Cur alleviated pathological changes in the liver and adipose tissues. Moreover, the ameliorative effect of the PE-Cur was more pronounced compared with free Cur.

### 3.6. PE-Cur Effectively Improved the Structure of the Gut Microbiota

**Figure 7:**
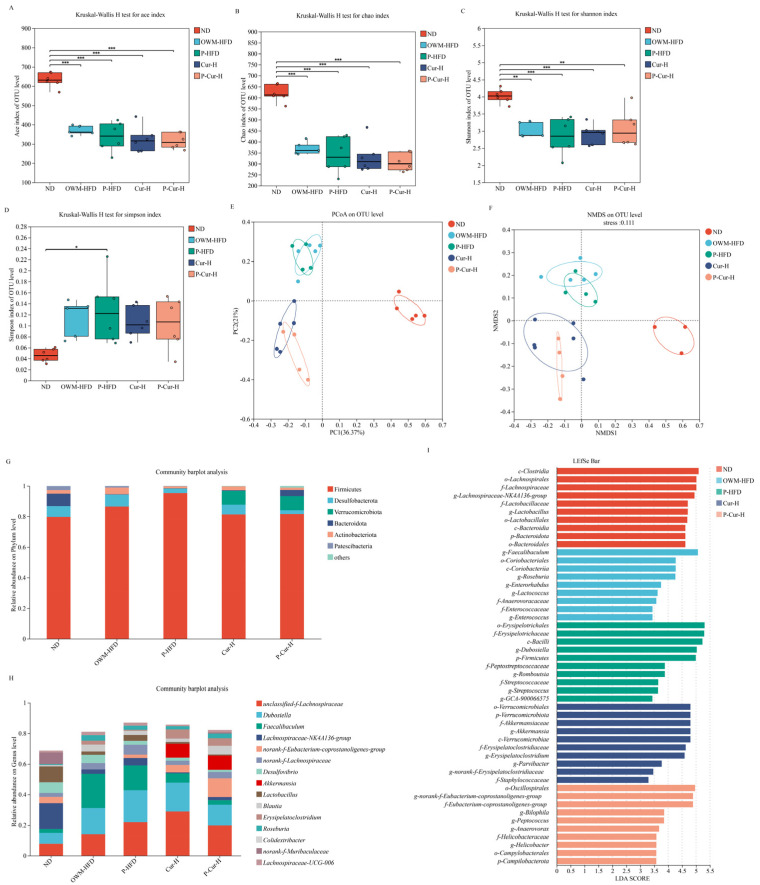
Changes in microbiota in the contents of the cecum of C57BL/J mice from different treatment groups (n = 6). (**A**) Ace Index; (**B**) Chao Index; (**C**) Shannon Index; (**D**) Simpson Index; (**E**) PCOA Principal Coordinate Analysis; (**F**) NMDS analysis; (**G**) gut flora at the portal level; (**H**) Intestinal flora at the genus level, “unclassified_f_” refers to taxa classified only at the family level according to the SILVA database; (**I**) abnormal enrichment of bacterial ASV levels in the intestines of mice from different treatment groups, LDA threshold > 2; * 0.01 < *p* ≤ 0.0.5, ** 0.001 < *p* ≤ 0.01, *** *p* ≤ 0.001.

Alpha diversity analysis showed that gut microbial richness and diversity significantly decreased in the HFD (OWM-HFD, P-HFD) groups and Cur intervention groups (Cur-HFD, P-Cur-HFD) relative to that in the ND group (Figure 7A–D, *p* < 0.05). The gut microbial composition showed distinct clustering in the ND, HFD, and Cur intervention groups.

As depicted in Figure 7G, on the phylum scale, the proportion of Firmicutes was 79.68% (ND), 86.56% (OWM-HFD), 95.26% (P-HFD), 81.32% (Cur-H), and 81.58% (P-Cur-H), respectively. The Bacteroidetes accounted for 8.09% (ND), 0.17% (OWM-HFD), 0.16% (P-HFD), 0.38% (Cur-H), and 3.92% (P-Cur-H), respectively. The relative abundances of Verrucomicrobiota were 0.03% (ND), 0.01% (OWM-HFD), 0.04% (P-HFD), 9.09% (Cur-H) and 9.30% (P-Cur-H), and those of the Desulfobacterota were 7.01% (ND), 7.82% (OWM-HFD), 2.96% (P-HFD), 6.35% (Cur-H), and 2.55% (P-Cur-H). At the phylum level, the HFD groups increased Firmicutes and decreased Bacteroidota. In the Cur intervention groups, the relative abundances of Firmicutes and Bacteroidota differed from those observed in the HFD groups. The Cur intervention groups showed higher Verrucomicrobiota and lower Desulfobacterota than the HFD groups. The PE-Cur group showed different relative abundances of Firmicutes, Bacteroidota, Verrucomicrobiota, and Desulfobacterota relative to the free Cur group.

At the genus level (as illustrated in Figure 7H), the ND group was dominated by Lachnospiraceae_NK4A136_group, *Lactobacillus*, and unclassified_f_ achnospiraceae. The dominant genera were *Faecalibaculum*, *Dubosiella*, and unclassified_f_Lachnospiraceae in the HFD groups. The Cur-HFD group featured Unclassified_f_Lachnospiraceae, *Dubosiella*, and *Akkermansia* as dominant genera. The P-Cur-HFD group was dominated by Unclassified_f_Lachnospiraceae, *Dubosiella*, and norank_f_Eubacterium_coprostanoligenes_group. Compared with the ND and HFD groups, the Cur intervention groups showed a significant increase in *Akkermansia* and a notable decrease in *Desulfovibrio*. This trend was more pronounced in the P-Cur-HFD group than in the Cur-HFD group, showing the stronger influence of PE-Cur on microbial composition.

Figure 7I showed marked increases in the ND (Clostridia and Lachnospirales), OWM-HFD (Faecalibaculum and Coriobacteriia), P-HFD (Erysipelotrichales and Erysipelotrichaceae), Cur-HFD (Verrucomicrobiales and Akkermansiaceae), and P-Cur-HFD (Oscillospirales and Eubacterium coprostanoligenes) groups.

### 3.7. The Effects of PE-Cur on SCFAs Levels

As shown in Table 1, the total content of SCFAs in the HFD groups (OWM-HFD, P-HFD) was reduced relative to the ND group (*p* < 0.05). Compared with the HFD groups, the Cur intervention groups (Cur-HFD, P-Cur-HFD) exhibited a higher total content of SCFAs (*p* < 0.05). The P-Cur-H group showed a higher SCFAs content than the Cur-H (*p* < 0.05). A similar pattern appeared in the changes of individual SCFAs, including acetic acid, propionic acid, isobutyric acid, butyric acid, isovaleric acid, valeric acid, ohexanoic acid, and hexanoic acid.

### 3.8. Correlation Analysis of Gut Microbiota-Serum Biochemical Factors and Gut Microbiota-SCFAs

**Figure 8:**
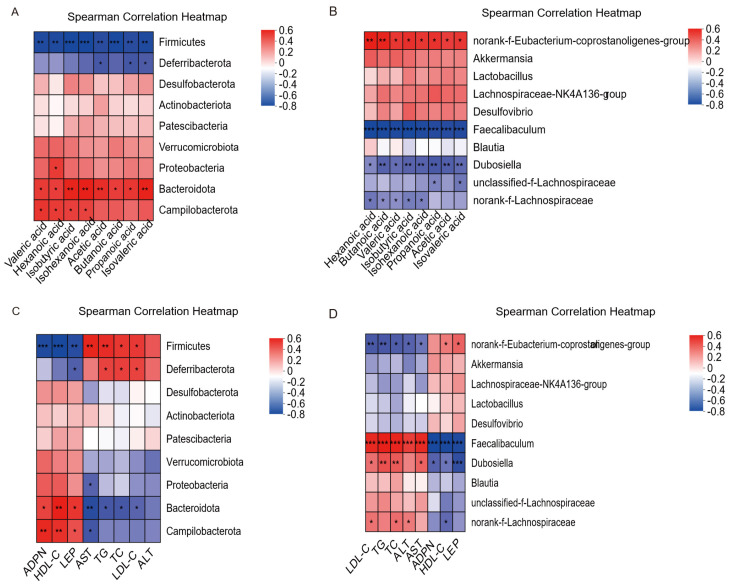
Correlation of gut microbiota with biochemical indices in serum and SCFAs in cecum contents. In serum (**A**) at the phylum level; (**B**) at the genus level; in cecum contents (**C**) phylum level; (**D**) genus level. Color shades indicate the degree of correlation (color scale: red = positive correlation; blue = negative correlation); * 0.01 < *p* ≤ 0.0.5, ** 0.001 < *p* ≤ 0.01, *** *p* ≤ 0.001.

As presented in Figure 8A, Firmicutes and Deferribacterota showed a negative correlation with ADPN, LEP, and HDL-C and were positively associated with AST, ALT, TC, TG, and LDL-C. The correlations between Firmicutes and ALT, as well as between Deferribacterota and ADPN, HDL-C, AST, or ALT, were not significant (*p* > 0.05). In contrast, Cyanobacteria, Verrucomicrobiota, Proteobacteria, Bacteroidota, and Campilobacterota were positively correlated with ADPN, LEP, and HDL-C and inversely correlated with AST, ALT, TC, TG, and LDL-C. No significant associations were found between Verrucomicrobiota and serum lipid-related parameters; between Proteobacteria and LEP, ALT, TC, LDL-C, or TG; between Bacteroidota and ALT or LDL-C; or between Campilobacterota and LEP or TC (*p* > 0.05). As Figure 8B showed, the norank-f-Eubacterium-coprostanoligenes-group was positively associated with ADPN, LEP, and HDL-C and inversely associated with AST, TC, ALT, TG, and LDL-C. Its correlation with ADPN was not significant (*p* > 0.05). *Faecalibaculum*, *Dubosiella*, and norank-f-Lachnospiraceae were inversely correlated with ADPN, LEP, and HDL-C and positively correlated with AST, ALT, TC, TG, and LDL-C. No statistically significant association was found between *Dubosiella* and ALT, nor between norank_f_Lachnospiraceae and ADPN, LEP, AST, ALT, or TG (*p* > 0.05). Based on Spearman correlation analysis, serum lipid metabolism-related parameters were correlated with gut microbial composition.

As shown in Figure 8C, Firmicutes and Deferribacteta were inversely correlated with SCFAs. The correlation between Deferribacterota and butyric acid, isobutyric acid, valeric acid, caproic acid, or isocaproic acid was not significant (*p* > 0.05). In contrast, Cyanobacteria, Bacteroidota, Campilobacterota, and Proteobacteria were positively correlated with SCFAs. The correlations of Campilobacteta with acetic acid and of Proteobacteria with acetic acid and butyric acid were not significant (*p* > 0.05). As presented in Figure 8D, norank_f_Eubacterium_coprostanoligenes_group, *Akkermansia*, *Lactobacillus*, Lachnospiraceae_NK4A136_group, and *Desulfovibrio* were positively associated with SCFAs, among which the norank_f_Eubacterium_ coprostanoligenes_group exhibited a significant correlation (*p* < 0.05). *Faecalibaculum, Dubosiella*, unclassified_f_Lachnospiraceae, and norank_f_Lachnospiraceae were also positively associated with SCFAs. The correlation between unclassified_f_Lachnospiraceae and isobutyric acid, acetic acid, valeric acid, butyric acid, or heptanoic acid was significant (*p* < 0.05), whereas the correlation between norank_f_Lachnospiraceae and acetic acid, propionic acid, or isopropyl caproic was not significant (*p* > 0.05). Spearman’s correlation test demonstrated a connection between gut microbiota composition and SCFAs production.

## 4. Discussions

HFD intake is a key contributor to LMDs. An LMD model was successfully established in C57BL/J mice using HFD. LMDs induced lipid accumulation in the mouse liver and adipose tissues. This subsequent lipid accumulation led to glucose intolerance, IR, and hepatic steatosis and ultimately resulted in obesity. This result aligns with the observations described by Sakamoto et al. [18]. HFD-induced glucose intolerance and IR are primarily attributed to multiple factors, including glucose transporter type 4 (GLUT4) dysfunction, sympathetic nerve activation [18], gut microbiota dysregulation [19], and LMDs [20]. HFD increases food intake in mice by downregulating the expression of silent information regulator 6 (SIRT6) in hypothalamic microglia [21]. Meanwhile, it promotes the binding of phosphorylated and activated activating transcription factor 2 (ATF2) to the promoter of peroxisome proliferator-activated receptor gamma coactivator 1-alpha (PGC-1α), reducing thermogenesis in brown adipose tissue [21]. These mechanisms lead to LMDs. LMDs are characterized by increased body weight, glucose intolerance, IR, and hepatic steatosis.

Several studies have revealed that Cur can effectively inhibit lipid synthesis and storage, stimulate fatty acid degradation, and thereby alleviate HFD-induced LMDs [6]. However, Cur has the limitation of low bioavailability due to its poor aqueous solubility and low stability [11]. Compared with other delivery systems, such as conventional nano-emulsions, liposomes, or nanocrystal suspensions, Pickering emulsions are stabilized by solid particles forming a densely packed and quasi-irreversible interfacial layer, which enhances the mechanical robustness of droplets and reduces coalescence during gastrointestinal transit [22]. The improved gastrointestinal stability and controlled release behavior of PE-stabilized curcumin nanocrystals were demonstrated, where the solid-particle interfacial layer provided protection against digestive disruption and enabled sustained release during intestinal transit, resulting in increased oral bioavailability [23]. A lot of studies in vitro have indicated that Cur encapsulated in PEs enhanced bioavailability [15,16]. In an in vitro study, the Cur bioavailability was enhanced from 19.53% to 41.26% following its encapsulation in PEs [16], while in vivo studies of PE-Cur on the regulatory effect of PE-Cur on LMD are unknown. The encapsulation efficiency of oil-in-water gel-based PEs containing sodium alginate (SA) is superior to those without SA due to their smaller droplets, stronger gel network, and higher crosslinking density [24]. Thus, in this study, Cur was encapsulated in oil-in-water gel-based PEs containing SA. The regulatory effect of the PE-Cur on HFD-induced LMDs and underlying mechanisms in vivo were investigated. The results demonstrated that, compared with free Cur, the PE-Cur more effectively alleviated HFD-induced LMDs in mice. These results can be partly attributed to the increased bioavailability of Cur in vivo after being encapsulated in PEs. The present study indicated that the PE-Cur significantly ameliorated the HFD-induced increases in body weight, liver index, and adiposity indices in mice, compared with free Cur. Meanwhile, compared with free Cur, PE-Cur significantly improved glucose tolerance and IR. Cur improves the abnormality of serum lipid metabolism-related parameters through multiple pathways. Cur reduces lipid biosynthesis by inhibiting the expression of fatty acid synthase and patatin-like phospholipase domain-containing protein 3 [25]. This further decreases hepatic fat accumulation and ultimately leads to a reduction in serum lipid metabolism parameters [25]. Cur can also improve serum lipid levels by adjusting gut microbiota-mediated bile acid homeostasis and activating Takeda G protein-coupled receptor 5 (TGR5) [19]. The present study showed that Cur intervention lowered serum TG, LDL-C, TC, ALT, or AST levels and elevated the HDL-C, ADPN, and LEP concentrations in HFD mice. These results are consistent with the findings of C. Yang et al. [26]. The present study indicated that compared with free Cur, there was a more significant improvement with PE-Cur. Simultaneously, Cur may promote glucose homeostasis and enhance insulin sensitivity through three mechanisms: first, it activates the phosphoinositide 3-Kinase/ protein Kinase B (PI3K/Akt) signaling pathway and increases threonine kinase phosphorylation and GLUT4 activity [25]; second, Cur reduces IR in peripheral tissues by improving β-cell function [27]; and third, it is possible that Cur enhances insulin sensitivity in HFD mice through upregulating FGF15 via gut microbiota [10]. These support that Cur could improve glucose intolerance and IR. This observation is consistent with the findings of the present study. The findings indicated that the ameliorative effect of PE-Cur is superior to that of free curcumin.

Cur reduced hepatic deposition and alleviated histopathological alterations in hepatic and adipose tissues. These results are consistent with the findings of Chiu, S. Li, et al. [9,28]. The ameliorative effect of PE-Cur was more significant compared with free Cur. Cur may alleviate hepatic steatosis through multiple pathways. In cholesterol metabolism, Cur inhibits intestinal cholesterol absorption and hepatic cholesterol synthesis by suppressing sterol regulatory element-binding protein-2 activity and downregulating the expression of Niemann-Pick C1-like 1 and 3-hydroxy-3-methylglutaryl coenzyme A reductase [29,30]. This further reduces hepatic cholesterol accumulation and lipogenesis levels [29,30]. In lipid regulation, Cur-mediated activation of PI3K/AKT phosphorylation enhances adipocyte glucose uptake, modulating adipogenesis and adipose tissue metabolic profiles [28]. In HFD-induced obese mice, Cur activates AMP-activated protein kinase (AMPK), which downregulates the lipid synthesis factor sterol regulatory element-binding protein 1 (SREBP-1) and upregulates peroxisome proliferator-activated receptor alpha (PPARα) associated with β-oxidation, further inhibiting hepatic lipid accumulation [31]. Additionally, Cur reduces hepatic accumulation through microbiota-mediated bile acid metabolism and activation of TGR5 [19]. Overall, Cur improves histopathological alterations in hepatic and adipose tissues through synergistic effects across multiple targets and pathways, including inhibiting lipid synthesis [29,30,31], promoting lipid catabolism [19], and remodeling gut microbial structure [19].

The present study demonstrated that Cur did not improve gut microbial richness and diversity, but exerted a remarkable effect on gut microbial composition. The results demonstrated that HFD, Cur, and PE-Cur influenced gut microbial composition. This finding is consistent with the results reported by Hong, S. Li, et al. [9,32]. Cur reduced the ratio of Firmicutes/Bacteroidota and enhanced Bacteroides abundance. Cur promoted beneficial gut microbiota (e.g., *Lachnospiraceae*, *Akkermansia*, and *Verrucomicrobiacea- e*) and reduced harmful *Desulfobacterota* in HFD mice. This outcome is in agreement with the results documented by Lamichhane, S. Li, T. Li, et al. [9,14,33]. Cur plays a significant role in modulating gut microbiota and improving related physiological functions. Cur can not only be metabolized by gut microbiota into highly active metabolites such as ferulic acid, tetrahydrocurcumin, demethoxycurcumin, and bisdemethoxycurcumin [34], but it can also regulate the gut microbial structure and maintain microbial homeostasis [34]. Cur has anti-inflammatory properties [33]. It alleviates gut microbiota imbalance-induced inflammation, reduces the level of the pro-inflammatorycytokine-IL-1β, and further enhances intestinal integrity [33]. Cur increases serum deoxycholic acid (DCA) level [19]. G protein-coupled receptor 5 is most strongly activated by DCA [19]. DCA activates the bile acid receptor TGR5, further regulating gut microbiota-modulated bile acid metabolism [19]. Regarding antioxidant activity and intestinal mucosal protection, Cur, as an effective electron donor, alleviates cellular damage and oxidative stress, protects the gut mucosa, and maintains intestinal flora homeostasis [35]. In energy metabolism, Cur enhances uncoupling protein 1-dependent thermogenesis by regulating gut microbiota, increasing energy expenditure, and reducing body weight and fat mass in mice [36]. Gut microbiota imbalance leads to IR and dysfunction in glucose and lipid metabolism [10]. Cur improves HFD-induced glucose intolerance, enhances insulin sensitivity, and alleviates LMD. This is achieved by regulating gut microbiota to upregulate the intestinal-derived hormone FGF15 [10]. Therefore, the amelioration of Cur on LMD may correlate with alterations in gut microbial composition. It exerts regulatory effects through multiple pathways and mechanisms.

SCFAs regulate lipid metabolism through enhancing insulin sensitivity, inhibiting fat production, and promoting liver fatty acid oxidation [37]. In addition, SCFAs, especially propionic acid and butyric acid, regulate LMDs by activating adenylate-activated protein kinase and peroxisome proliferator-activated receptor γ (PPARγ), promoting fatty acid oxidation and inhibiting lipid synthesis [38]. Cur achieves metabolic regulation via reshaping the structure of gut microbiota [19]. Cur reduces the Firmicutes-to-Bacteroidetes ratio and elevates the proportion of SCFAs-producing *Bacteroidetes* [19]. Therefore, Cur may regulate LMD through regulating gut microbial composition and influencing the metabolic products SCFAs production. The PE-Cur exhibited more pronounced changes than the Cur. The main reason is that the stability and intestinal absorption rate of Cur encapsulated in PE are significantly improved [14], further enhancing the ability of regulating the microbiota metabolism and efficiently promoting the metabolic activity of SCFAs-producing microbiota.

The dominant microbiota altered by Cur was closely associated with the serum lipid-related metabolic indicators in HFD mice [19]. As the main metabolites of dietary fiber after fermentation by gut microbiota, SCFAs improve the stability of the intestinal microecology [39]. Multiple bacterial groups show a high degree of correlation with SCFAs [40]. This study clarified that HFD significantly reduced SCFAs in the cecal contents, whereas Cur effectively reversed this trend. Compared with free Cur, the PE-Cur exhibited a more pronounced promoting effect on SCFAs production in mice intestines.

Cur showed a multidimensional improvement effect on HFD-induced LMD. Cur not only regulated serum lipid levels, alleviated glucose intolerance, improved IR, and alleviated fatty liver degeneration, but also maintained the gut microbiota balance and promoted SCFA production in the gut. In conclusion, Cur most likely regulates LMD via reshaping the gut microbial composition and further regulating the production of metabolic SCFA production. Cur-mediated regulation of LMDs was closely associated with gut microbiota remodeling and the production of its metabolite, SCFA. Compared with free Cur, PE-Cur exerted a more pronounced effect on HFD-induced LMDs, which provided an effective approach for the delivery of Cur. In the future, the tissue-distribution of PE-Cur can be studied, which can further confirm the metabolic absorption pathway of PE-Cur after it enters the body.

## 5. Conclusions

This study demonstrated that curcumin encapsulated in Pickering emulsions effectively alleviated high-fat-diet-induced obesity and lipid metabolism disorders in C57BL/6J mice. The curcumin encapsulated in Pickering emulsions exerted these effects by regulating the levels of lipid-related parameters in serum, reducing high-fat-diet-induced lipogenesis, alleviating glucose intolerance, improving insulin resistance, restoring gut dysbiosis, and modulating the metabolic products short-chain fatty acids. Compared with free curcumin, curcumin encapsulated in Pickering emulsions has a more significant improvement effect on high-fat-diet-induced lipid metabolism disorders. This study provides an effective strategy for the delivery of curcumin, highlighting the great potential of curcumin encapsulated in Pickering emulsions as a natural alternative for preventing high-fat-diet-induced hepatic steatosis. Further clinical investigations are necessary to validate its efficacy and explore its potential application as a functional food.

## Figures and Tables

**Table 1:** The effect of curcumin on short-chain fatty acids in the cecal contents of mice.

Aecal Digesta (μg/100 mg)	ND	OWM-HFD	P-HFD	Cur-H	P-Cur-H
Acetic acid	242.19 ± 1.95 ^a^	169.35 ± 4.34 ^d^	165.28 ± 3.88 ^d^	194.27 ± 3.95 ^c^	224.06 ± 4.60 ^b^
Propanoic acid	59.03 ± 1.92 ^a^	41.88 ± 1.89 ^d^	39.92 ± 1.88 ^d^	48.13 ± 2.57 ^c^	54.16 ± 1.80 ^b^
Isobutyric acid	6.76 ± 0.15 ^a^	5.49 ± 0.24 ^d^	5.40 ± 0.19 ^d^	6.11 ± 0.17 ^c^	6.44 ± 0.11 ^b^
Butanoic acid	50.45 ± 1.42 ^a^	41.86 ± 1.47 ^d^	40.61 ± 1.24 ^d^	45.66 ± 0.83 ^c^	48.86 ± 1.02 ^b^
Isovaleric acid	6.60 ± 0.23 ^a^	5.25 ± 0.18 ^d^	5.07 ± 0.15 ^d^	6.00 ± 0.10 ^c^	6.23 ± 0.12 ^b^
Valeric acid	8.27 ± 0.19 ^a^	7.36 ± 0.16^d^	7.22 ± 0.13 ^d^	8.00 ± 0.10 ^c^	8.13 ± 0.06 ^ab^
Isohexanoic acid	1.47 ± 0.13 ^a^	0.79 ± 0.02 ^d^	0.66 ± 0.02 ^c^	1.09 ± 0.11 ^b^	1.17 ± 0.11 ^b^
Hexanoic acid	0.30 ± 0.01 ^a^	0.24 ± 0.01 ^c^	0.22 ± 0.01 ^c^	0.28 ± 0.01 ^b^	0.31 ± 0.01 ^a^
Total SCFAs	375.08 ± 1.00 ^a^	272.20 ± 1.39 ^b^	264.38 ± 1.25 ^c^	309.54 ± 1.31 ^d^	349.36 ± 1.31 ^e^

Data were expressed as mean ± RSD. Each group is *n* = 6. Values in the same row marked with different letters are significantly different at *p* < 0.05.

## Data Availability

The data presented in this study are available on request from the corresponding author. (The data are not publicly available due to ethical and institutional restrictions related to animal experimentation).

## Abbreviations

LMDs: Lipid metabolism disorders; Cur: Curcumin; PEs: Pickering emulsions; PE-Cur: Cur encapsulated in Pickering emulsions; HFD: High-fat diet; IR: Insulin resistance; SCFAs: Short-chain fatty acids; FGF15: Fibroblast growth factor 15; ND: Normal diet group; OWM-HFD: HFD supplemented with oil-water mixture group; P-HFD: HFD supplemented with the oil-in-water gel PE group; Cur-HFD: HFD supplemented with oil-water mixture containing Cur group; P-Cur-HFD: HFD supplemented with the oil-in-water gel PE encapsulating Cur group; BAT: Brown adipose tissue; iWAT: Inguinal white adipose tissue; eWAT: Epididymal white adipose tissue; PFA: 4% paraformaldehyde solution; OGTT: Oral glucose tolerance test; ITT: Insulin tolerance test; AUC: The area under the curve; TG: Triglycerides; TC: Total cholesterol; LDL-C: Low-density lipoprotein cholesterol; HDL-C: High-density lipoprotein cholesterol; ALT: Alanine aminotransferase; AST: Aspartate aminotransferase; ADPN: Adiponectin; LEP: Leptin; RSD: Relative standard deviation; ANOVA: One-way analysis of variance; PCOA: Principal coordinate analysis; NMDS: Non-metric multidimensional scaling; LEFSE: Linear discriminant analysis effect size; GLUT4: Glucose transporter type 4; SIRT6: Silent information regulator 6; ATF2: Activating transcription factor 2; PGC-1α: Peroxisome proliferator-activated receptor gamma coactivator 1-alpha; SA: sodium alginate; TGR5: Takeda G protein-coupled receptor 5; PI3K/Akt: Phosphoinositide 3-Kinase/protein Kinase B; AMPK: AMP-activated protein kinase; SREBP-1: Sterol regulatory element-binding protein 1; PPARα: Peroxisome proliferator-activated receptor alpha; IL-1β: interleukin-1β; DCA: Deoxycholic acid; PPARγ: Peroxisome proliferator-activated receptor γ.
